# Lomb’s Prize Essays

**Published:** 1888-06

**Authors:** 


### Lomb's Prize Essays. Published by the American Public Health Association.

Mr. Henry Lomb has given the Public Health Association a fund to defray the expenses of publishing tracts upon hygiene, etc., for distribution among the poorer classes at a nominal cost. The idea is a noble and useful one, as it strives to render the poor healthier and happier.

The final outcome of Mr. Lomb's generous offer, and of the action of the several Committees of Award appointed by the Association, has been the publication of four valuable treatises on the following subjects:

No. 1. Healthy Homes and Foods for the Working Classes. 10 cents. By Professor Victor C. Vaughan, M. D., Ann Harbor, Mich.

No. 2. The Sanitary Conditions and Necessitifs of School-Houses and School Life. 5 cents. By D. F. Lincoln, M. D., Boston, Mass.

No. 3. Disinfection and Individual Prophylaxis against Infectious Diseases. 5 cents. By George M. Sternberg, M. D., Major and Surgeon U. S. A.

No. 4. The Preventable Causes of Disease, Injury, and Death in American Manufactories and Workshops, and the Best Means and Appliances for Preventing and Avoiding Them. 5 cents. By George H. Ireland, Springfield, Mass.

